# Low coverage of intermittent preventive treatment for malaria in pregnancy in Nigeria: demand-side influences

**DOI:** 10.1186/1475-2875-11-82

**Published:** 2012-03-23

**Authors:** Chima A Onoka, Kara Hanson, Obinna E Onwujekwe

**Affiliations:** 1 Health Policy Research Group, College of Medicine, University of Nigeria, Enugu campus, Enugu, Nigeria; 2 London School of Hygiene and Tropical Medicine, London, UK; 3 Department of Community Medicine, College of Medicine, University of Nigeria, Enugu, Nigeria; 4 Department of Pharmacology and Therapeutics, College of Medicine, University of Nigeria, Enugu, Nigeria

**Keywords:** Malaria, Intermittent Preventive Treatment in Pregnancy, Sulphadoxine-Pyrimethamine, Coverage, Nigeria

## Abstract

**Background:**

Nigeria instituted intermittent preventive treatment for malaria (IPTp) using sulphadoxine-pyrimethamine (SP) for pregnant women in 2001, but coverage remains low. This study examined the influence of demand side factors on IPTp coverage.

**Methods:**

Data were collected using a household survey from 1307 women who were delivered of a live baby within the one-year period preceding the study and through an exit poll from 146 women attending antenatal clinics (ANC). Data analysis examined coverage based on the national and WHO guidelines for IPTp delivery which differ with regards to use of IPTp in the last month of pregnancy. Focus group discussions (FGDs) were undertaken to further explain demand side constraints to coverage.

**Results:**

From the household survey, 96.1% of respondents attended ANC, with most having five or more visits. Overall IPTp coverage for the first and second doses was 13.7% and 7.3% respectively. The coverage was higher in the urban areas compared to rural areas (p < 0.01). Amongst women who could have received IPTp based on the timing of their attendance, only 14.1% and 14.3% were offered the first dose based on national and WHO guidelines, while 7.7% and 7.5% were offered the second dose respectively giving significant missed opportunities. Amongst ANC attendees offered first and second doses, 98.9% and 96.9% respectively took the medicine. Only 13.6% and 21.5% of these clients reported taking the drug under direct observation. The low level of coverage was confirmed by exit survey data, which found coverage of 11.6% and 3.0% for the first and second doses. The FGDs revealed that women do not have many concerns about side effects, but they take drugs providers give them because they believe such drugs must be safe.

**Conclusion:**

This study found low coverage of IPTp and high levels of missed opportunities supporting findings that high ANC attendance does not guarantee high IPTp coverage. Demand side factors such as ANC attendance, appropriate timing of attendance, and perceptions about side effects were not constraining factors to increased coverage, raising the need to examine supply side factors.

## Background

Malaria contributes to 11% of maternal deaths in Nigeria [1]. Parasite prevalence in pregnant women in Nigeria could be as high as 60-70% [2]. These women are infected with *Plasmodium falciparum*, the most virulent Plasmodium with serious health consequences including anaemia, impaired foetal growth, still birth, and premature delivery [3,4].

Intermittent-preventive treatment of malaria in pregnancy (IPTp) using sulphadoxine-pyrimethamine (SP) was approved by the World Health Organization (WHO) Expert Committee on Malaria for the control of malaria in pregnancy in areas of moderate to high transmission [5]. This followed a number of studies documenting its effectiveness [4,6,7]. SP was also found to be effective in increasing birth weight and reducing prevalence of preterm deliveries and maternal anaemia in Nigeria [8].

In May 2000, African leaders in Abuja under the Roll Back Malaria (RBM) partnership set the target that by 2005 at least 60% of all pregnant women who are at risk of malaria, especially those in their first pregnancies should have access to chemoprophylaxis or IPT [9]. The WHO reports that at the end of 2008, 35 of 45 sub-Saharan African countries had adopted IPTp as national policy [10]. However, coverage has remained far from the target in many countries including Nigeria.

In 2001, Nigeria instituted intermittent preventive treatment using SP for pregnant women in the second and third trimesters of pregnancy. However, both first and second dose coverage remains low being 8.0% and 4.6% respectively in Nigeria and 9.9% and 5.4% in south-east Nigeria [11]. The use of IPTp in Nigeria involves the administration of at least two curative doses of SP during pregnancy, regardless of whether the woman is infected [12]. The first dose is taken after quickening (WHO 2004) and there should be at least one month between the two doses. However, although the WHO guideline allows the administration of IPTp all through the third trimester, the policy in Nigeria recommends otherwise, stating that *'the last dose should be given not later than one month before the expected date of delivery' *[12]. Direct observed treatment (DOT) by a qualified health worker was also incorporated to ensure compliance by pregnant women [13]. Compliance is further enhanced by the single dose treatment of SP.

Successful deployment of IPTp is dependent on the utilization rates of antenatal care (ANC) services amongst pregnant women. Attendance at ANC is high in most sub-Saharan African countries [5], but up to 25% of pregnant women pay the first visit in the 3 rd trimester [14]. This may affect the impact of ANC and IPTp related services as delivery of the second dose of SP is substantially reduced and envisaged protection for mother and foetus is lost [15].

A woman that attends ANC needs to do so at appropriate times for delivery of IPT, which is best given when the growth of the foetus is occurring at its highest velocity (16th - 24th week) as this helps to reduce placental parasitaemia, foetal growth reduction and the resultant low birth weight [16]. Whether a woman starts early or late, each visit should count so that opportunities created by her attendance to ANC are not missed for the delivery of relevant interventions. However, studies have shown that missed opportunities abound and constitute a challenge for IPTp delivery [17,18].

Apart from failure to attend ANC clinics, other identified barriers to use of IPTp include poor acceptance of SP because of perceived association of SP with side effects, abortions and foetal deformities [19-21]. However, clinical studies have shown such side effects are uncommon [4,6,7,22].

This study examined whether demand side factors constrain coverage of IPTp amongst pregnant women. This enabled the identification of opportunities to intervene to improve coverage. The study also examined the differences in coverage and demand side barriers between urban and rural dwellers. These are lacking for Nigeria where maternal mortality remains high[11].

## Methods

### Study area

This study was carried out in 2010 in Enugu State, south-east Nigeria. Enugu state has 17 Local Government Areas (LGAs), a total population of 3,257,298 and an annual population growth rate of 3.0 [23]. Enugu North and South LGAs (Enugu), which have a combined population of 443,575 were purposively selected to represent urban areas while Udi LGA which has a population of 234,002, was selected to represent rural areas. Udi has 17 public primary health facilities that offer ANC services while Enugu has 11. The communities used were selected by simple random sampling from a list of communities in Enugu and Udi.

### Data collection

A household and a facility exit survey were used to elicit information on coverage, demand side factors that could explain the level of coverage observed, and the practice of directly observed treatment with IPTp. In addition, focus group discussions (FGDs) were undertaken to further provide qualitative data on demand side constraints to coverage.

#### Household survey

A pre-tested interviewer-administered questionnaire was used to elicit information from a total of 1307 women who had pregnancies with live births as outcome in the one-year period preceding the study. The minimum sample size required was computed separately for the urban and rural areas based on a 95% confidence level and an IPTp coverage of 12.6% and 6.0% in urban and rural areas of Nigeria, respectively [11]. For each LGA, three out of eight communities were initially selected by simple random sampling. In the second stage, women within each community were initially listed following visits to their households and a constant proportion of women from the sampling frame of each community was randomly selected to ensure a self weighted sample.

#### Exit poll

All the 28 primary health care facilities providing antenatal care in the study sites were used. A pre-tested interviewer administered questionnaire was used to collect information from 146 women that had completed 8 months of pregnancy, and so were no longer eligible to receive IPTp based on the national guidelines. The minimum sample size required was determined based on a confidence level of 95%, an error margin of 0.05, and an IPTp coverage level of 8% for women receiving antenatal care from a skilled provider in the southeast region [11]. Since the level of attendance of ANC varies for different facilities, a proportionate method was used for determining the sample size for each facility. The sample for each facility was determined by weighting the total sample size required with the relative proportion of clients that the facility handles - using the total attendance figures for each facility (as reported by the heads of the facilities) for the week prior to commencement of data collection as numerator, and the sum of ANC attendees in all the facilities as denominator. Half of the required sample for each facility was selected on each of the two days that facilities run ANC clinics in a week from the group of women attending the compulsory general health talk organized for all women with ANC clinic appointments for the day.

#### Focus group discussion

Focus group discussions (FGDs) were held with women of childbearing age (15-49 years) who delivered live babies within the one-year period preceding the study. Four FGDs were held - one for each of the four study sites. There were 8-10 members in each group and each FGD lasted between 45 and 60 minutes. The participants were purposively selected with the help of a community contact person, so that all sections of each community were represented. A topic guide, focusing on awareness about IPTp delivery and factors influencing its uptake by women, was used to guide the discussions and an electronic voice recorder was used for the interviews.

### Ethical considerations

Ethical clearance was obtained from the Research Ethics Committees of the University of Nigeria Teaching Hospital Enugu and the London School of Hygiene and Tropical Medicine, while permission to carry out the study was obtained from the Primary Health Care Coordinator of each of the LGA's and from the heads of facilities used. All respondents and interviewees were required to sign or thumb-print a consent form after they had been informed of the objectives of the study and the voluntary nature of their participation.

### Data analysis

#### Quantitative data

Data was pooled within the urban and rural areas. Coverage was measured by asking standard questions about IPTp used for Demographic and Health Survey data [11]. Coverage of IPTp was analysed in three dimensions: based on all respondents (overall coverage), based on all respondents that attended ANC, and based on respondents that made "timely attendance" or "timely visit" defined here as *attendance to ANC at a time a pregnant woman should be offered IPTp based on existing guidelines for IPTp delivery*. In order to assess "timely attendance", respondents were categorized into groups by matching the information about the gestational age of pregnancy at the times they made ANC visits with the national [1] and WHO [5] guidelines for timing of delivery of IPTp. Based on the national guideline, a pregnant woman should not be offered IPT too early (before quickening - before or during the 4^th ^month) or too late (within the last month of pregnancy), and needs a gap of one month between two doses. Attendance to ANC between these early and late points was defined as "timely attendance". For the WHO guidelines, the period considered as "timely attendance" extends through the third trimester. In the case of the WHO guidelines more women would be expected to meet the inclusion criterion because the guideline does not restrict IPTp delivery within the last month of pregnancy. As shown in Table 1, categories B-D represent those that should be offered the first dose while only those in category C should be offered a second dose. Missed opportunities included women who made timely visits but were not offered IPTp [17,18]. Data were analysed in STATA version 11 using the 'svyset' commands in order to adjust for the cluster survey design. The communities/health facilities were used as the primary sampling units and urban/rural area as 'strata'.

**Table 1:** Categories for timely attendance

Category Description of category		Theoretical implications of timing and adequacy of visits for ANC
**A**	No visit within appropriate time	*Should not be offered SP*

**B**	Only one timely visit	*Can only receive one dose. The later the visit, the less the period of protection*

**C**	Made at least 2 timely visits that were at least one month apart	*Can be offered 2 doses of IPTp. Early attendees (those within 5^th ^month) achieve the best protection*

**D**	Up to 2 visits within appropriate time but visits were less than one month apart to receive 2 doses	*Should only receive 1 dose. Period of protection is also affected by the timing of the earliest visit*

#### Qualitative data analysis

The records of FGDs were transcribed and responses were categorized into domains representing common themes. The areas of consensus and divergence in the responses according to the groups and study areas were determined for better identification of influences constraining coverage. Content analysis was used to categorize responses into domains representing common themes.

## Results

The average age of respondents in the household survey was 28.3 years (Standard error = 0.11) (Table 2). Most of the women completed secondary education with the proportion slightly higher in the urban area. In both LGAs, most respondents were married. Similarly, the mean age of respondents for the exit interview was 28.4 years (SE = 0.83); most of them completed secondary school and were married.

**Table 2:** Socio-demographic characteristics of respondents

Variable	Household survey			Exit interview
	
	URBAN	RURAL	Total	N = 146
	N = 806	N = 501	N = 1307	%
	%	%	%	
**Age: mean (standard error)**	28.4 (0.13)	28.3(0.19)	28.3 (0.11)	28.4 (0.83)

**Highest Level of education reached**				
None	0.4	1.0	0.6	0.7
Incomplete Primary	0.7	2.0	1.2	2.1
Complete Primary	8.3	10.2	9.0	9.6
Secondary +	90.6	86.8	89.1	87.7

**Current marital status**				
Single	3.0	4.0	3.4	4.8
Married	91.2	88.4	90.1	88.4
Widowed	5.3	5.6	5.4	6.2
Divorced/separated	0.5	2.0	1.1	0.7

### Antenatal care attendance and coverage of IPTp amongst survey respondents

IPTp coverage amongst all respondents for the first dose was 13.7% (16.9% in urban areas and 8.6% in rural areas, p < 0.01) [Table 3]. Second dose coverage was 7.3% and higher as well for the urban area though not statistically significant. Most women interviewed in the household survey (96.1%) attended antenatal care at least once, with significantly higher attendance among urban dwellers (99.3%) than rural dwellers (91%) [p < 0.005]. The majority of women made five or more visits for ANC, though the share was higher for urban than rural areas (89.2% vs. 78.7%, p < 0.001). Amongst ANC attendees, 1^st ^dose coverage was 14.3% while 2^nd ^dose amongst 1235 respondents that made at least two ANC visits was 7.8%.

**Table 3:** Coverage of IPTp for malaria amongst respondents

		Household Survey		P*	Exit Poll
	**URBAN**	**RURAL**	**Total**		
	**N = 806**	**N = 501**	**(N = 1307)**		**N = 146**
	**n(%) [CI]**	**n(%) [CI]**	**n(%) [CI]**		**n(%) [CI]**

**IPTp Coverage and ANC attendance amongst all respondents**					

First dose	136 (16.9) [15.2-18.7]	43 (8.6) [5.3-13.4]	179 (13.7) [12.0-15.5]	0.009*	
Second dose	71 (8.8) [7.3-10.6]	25 (5.0) [2.5-9.6]	96 (7.3) [5.9-9.8]	0.085	
Attended ANC at least once	800 (99.3) [96.7-99.8]	456 (91.0) [89.0-92.7]	1256 (96.1) [95.1-96.9]	0.001*	
Attended ANC ≥ 2 times	785 (97.4) [94.8-98.7]	450 (89.8) [87.7-91.6]	1235 (94.5) [92.9-95.8]	0.002*	132 (90.4) [79.3-95.9]

**Coverage amongst ANC recipients**					

***First dose amongst those attending at least once***					
- Offered dose	136 (17.0) [15.3-18.8]	43 (9.4) [6.0-14.6]	179 (14.3) [12.6-16.1]	0.015*	17 (11.6)[5.3-25.6]
- Took drug	135 (98.9) [94.6-99.9]	42 (97.7) [84.2-99.7]	177 (98.9) [94.6-99.8]	0.383	17 (100)
***Second dose amongst those attending ***					
- Offered dose	71 (9.0) [7.6-10.7]	25 (5.6) [2.8-10.8]	96 (7.8) [6.3-9.6]	0.134	4 (3.0) [0.9-9.3]
- Took drug	70 (98.6) [88.3-99.9]	23 (92.0) [63.6-98.7]	93 (96.9) [86.1-99.4]	0.144	4 (100)

*P values are for tests of significance for the difference in proportions between urban and rural subgroups. Differences are statistically significant if p < 0.05.

Amongst all the women that received antenatal care, 97.3% and 91.5% paid timely visits for the first dose and up to two doses of IPTp respectively based on the national guidelines (Table 4). Of these women, 14.1% and 7.7% were offered the first and second doses respectively giving missed opportunities of 85.9% and 92.3%. More urban dwellers were offered the first dose (p < 0.01). Based on WHO guidelines, 99.5% and 95.8% paid timely visits for the first and up to two doses, respectively, while 14.3% and 7.5% were offered IPTp. Seven and seven respondents who attended at inappropriate times for IPTp were offered the first and second doses respectively based on the national guidelines. However, all sevens offered the first dose and one of those offered the second dose were eligible based on the WHO guidelines. Additional 28 and 54 women, for first and second doses, respectively, would have also been eligible for these doses based on the WHO guidelines.

Amongst ANC attendees offered first and second doses, 98.9% and 96.9% accepted the medicine (Table 3). The two respondents who did not take the first dose felt it would affect the baby while all the three that did not take the second dose felt that the time was too close to delivery for them to take it.

For the exit survey respondents, overall IPTp coverage amongst all ANC attendees was 11.6% for the first dose and 3.0% for the second dose amongst those attending at least two times (Table 3). Based on the national guidelines, the figures for timely attendance for the first and second doses were 93.8% and 76.5% respectively, and the coverage amongst them was 12.4% and 4.0%, giving a missed opportunity of 87.6% and 96.0% respectively (Table 4). All those that received the first and second doses took them.

**Table 4:** Coverage amongst ANC recipients that paid timely visits based on national and WHO guidelines

		Household Survey		P*	Exit Poll
	**n (%) [CI]**	**n (%) [CI]**	**n (%) [CI]**		**n (%) [CI]**

***First dose***					
- Timely attendance	776 (97.0) [95.4-98.1]	446 (97.8)	1222 (97.3) [95.7-98.3]	0.593	137 (93.8) [88.9-96.6]
- Offered dose	131 (16.9) [15.4-18.5]	41 (9.2) [6.0-13.8]	172 (14.1) [12.6-15.7]	0.009*	17 (12.4) [5.6-25.2]
- Missed opportunity	645 (83.1) [81.5-84.6]	405 (90.8) [86.3-94.0]	1050 (85.9) [84.3-87.4]		120 (87.6) [74.8-94.4]

***Second dose***					
- Timely attendance	731 (91.4) [88.9-93.3]	418 (91.7) [87.8-94.4]	1149 (91.5) [89.5-93.1]	0.865	101 (76.5) [69.2-82.6]
- Offered dose	67 (9.2) [7.9-10.6]	22 (5.3) [2.9-9.5]	89 (7.7) [6.4-9.3]	0.066	4 (4.0) [1.2-12.4]
- Missed opportunity	664 (90.8) [89.4-92.2]	396 (94.7) [90.5-97.2]	1060 (92.3) [90.7-93.6]		97 (96.0) [87.6-98.8]

**Coverage amongst ANC recipients that paid timely visits based on WHO guidelines**					

***First dose***					
- Timely attendance	795 (99.4) [98.4-99.8]	455 (99.8) [97.8-99.9]	1250 (99.5) [98.9-99.8]	0.334	
- Offered dose	136 (17.1) [15.4-18.9]	43 (9.5) [6.0-14.6]	179 (14.3) [12.6-16.2]	0.015*	
- Missed opportunity	659 (82.9) [81.1-84.6]	412 (90.6) [85.4-94.0]	1071 (85.7) [83.8-87.4]		

***Second dose***					
- Timely attendance	765 (95.6) [93.6-97.0]	438 (96.1) [90.2-98.5]	1203 (95.8) [93.7-97.2]	0.817	
- Offered dose	68 (8.9) [7.4-10.6]	22 (5.0) [2.7-9.2]	90 (7.5) [6.1-9.1]	0.066	
- Missed opportunity	697 (91.1) [89.4-92.6]	416 (95.0) [90.8-97.3]	1113 (92.5) [90.9-93.9]		

*P values are for tests of significance for the difference in proportions between urban and rural subgroups. Differences are statistically significant if p < 0.05.

### Source of drug for IPTp and practice of directly observed treatment amongst those who took the drug

Of the household survey respondents who took the drugs for IPTp, 62.7% and 58.1% obtained the first and second doses free at the health facility respectively (Table 5). Only 13.6% and 21.5% of these patients reported taking the first and second doses offered at the facility under direct observation. For the exit poll, 13 (76.5%) of the 17 respondents that took the first dose of SP obtained it free at the facility while the rest purchased it at the facility. However, 82.4% took it at home, while the rest took it in the facility under observation. Similarly, three out of the four that took the second dose did so at home while one took it at the facility under observation. Two of them obtained it at the facility while the rest purchased it at the facility.

**Table 5:** Source of drug for IPTp and practice of directly observed treatment amongst recipients

	1^st ^Dose			2^nd ^Dose		
	**URBAN**	**RURAL**	**Total**	**URBAN**	**RURAL**	**Total**
	**N = 136**	**N = 43**	**N = 179**	**N = 71**	**N = 25**	**N = 96**
	**%**	**%**	**%**	**%**	**%**	**%**

**How drug was obtained**						
Received free at the health facility	60.0	71.4	62.7	52.9	73.9	58.1
Bought drug at health facility	38.5	26.2	35.6	44.3	26.1	39.8
Bought drug elsewhere	1.5	2.4	1.7	2.9	0	2.2

**Where drug was taken**						
In facility under observation	9.6	26.2	13.6	17.1	34.8	21.5
In facility without observation	1.5	2.4	1.7	1.4	0	1.1
At home	88.9	71.4	84.7	81.4	65.2	77.4

### Results of focus group discussions

Only two out of all participants had ever heard of IPTp. One (from the urban area) heard of it when some visitors to the facility she registered talked about it. The second woman (from the rural area) heard of it from a friend in school. Thus, no one heard of IPTp from health workers. The two who said they knew about it said it was *'something they do to prevent malaria' *but had no idea what drug is used, how many doses a woman should take, when in pregnancy it should be given and how it should be given. The participant from the rural area however reported that a woman that books at the eight month of pregnancy should not receive two doses.

After IPTp was described to participants, some participants from one of the four discussion groups (rural area) recalled being given SP (in combination with other drugs) by health workers during pregnancy for prevention, but not for treatment. Two recalled being asked by the caregiver to take two tablets first and one later while another took three at a time and was given a second dose one month before delivery.

Concerning use of SP during pregnancy, participants did not see any cause for worry about use of the drug during pregnancy. Side effects could happen but *'this is seen in all drugs used for malaria.' *Rather, women are worried when they do not know the drugs to take; but they take drugs health workers give them because '*they *(the health workers) *will only give the safe ones*.' They also did not identify any existing cultural factor that hinders them from going for antenatal care or taking drugs given to them by health workers. The only concern expressed by participants in the rural area was that when women are taking herbal drugs given to them (usually by the TBA or mothers), they collect and keep the 'modern drug' and may have to wait to finish the herbal one before taking drugs given to them by antenatal care providers. Husbands, mothers of pregnant women and their mothers-in-law were mentioned as the only people who could influence the decision of a woman to take the drug or not. Even at that, participants did not consider their influence significant.

## Discussion

The level of coverage of IPTp in the study area was low despite the fact that a policy has been in existence in the country for a decade. However, the first dose coverage that was found in this study (13.7%) was significantly higher than that reported in the DHS 2008 for the southeast region of the country (*χ*^2 ^= 7.96, p < 0.05), suggesting that further improvements have been made in coverage in the region. However, there was no significant difference for the second dose coverage found when compared with 5.4% coverage documented in the DHS 2008 (*χ*^2 ^= 3.8, p > 0.05).

It is worthy of note that first dose coverage was lower amongst women living in rural areas compared to their urban counterparts. Earlier reports found coverage levels of 12.6% and 6.0% amongst women living in urban and rural areas of the country, respectively [11]. This unequal coverage indicates that access barriers still exist to higher degrees for those in the rural areas.

The overall low coverage occurs despite the finding that nearly all women receive antenatal care and most make enough visits. Higher IPTp coverage has been reported even in settings with lower ANC attendance rates [24]. Other authors note that high attendance to ANC does not translate to high IPTp coverage [15,25]. This study shows that women pay enough visits for ANC and should be eligible to receive IPTp. Moreover, even though they do not know enough about IPTp to make informed demand for it, they make themselves available enough to receive interventions provided at the facility.

Apart from making the right number of visits, the visits should be appropriately timed. This study shows that not only did women go for ANC, most women attended at appropriate times for the delivery of two doses of IPTp irrespective of the guideline used for examining attendance. Attendance to ANC was timely for up to two doses to be offered even when they paid the first visit late in their pregnancy. Further still, geographic location did not affect timely attendance amongst ANC attendees. Yet, even amongst women who paid timely visits, the coverage was still very low. Similar observations have been made elsewhere [26]. What abound are missed opportunities, which, if taken advantage of, provide enough opportunities for increase in coverage. This finding suggests that supply rather than demand side factors could be responsible for low coverage of IPTp and need to be examined. The problem of missed opportunities is further compounded by another supply related issue - the national guidelines - which make women ineligible for IPTp during the last month of pregnancy, even though such a practice is not supported by clinical evidence [5].

A common concern that may affect use of IPTp is that women may be unwilling to take drugs given to them, usually because of side effects [19]. This study however found that women take drugs given to them once it comes from the health workers, and do not have serious concerns about side effects which may affect them. Additionally, they do not think cultural barriers affect their demand for IPTp. This suggests that demand side concerns are unlikely to be responsible for low coverage and use of IPTp.

The study found that over three-quarter of those that took drugs reported that they were allowed to take the drug home, contrary to the guidelines for IPTp administration which stipulate the use of DOT. The findings of the exit interview corroborate those of the household survey and similar reports of poor experience of DOT elsewhere [19,27]. The implications for effectiveness of deployment of the intervention are significant - women are poorly covered with the intervention, and for the few who receive it, its effectiveness is likely to be further hampered by poor utilization practices which conflict with recommended guidelines. It would be useful to determine why limited use of DOT is reported by the women by exploring supply side practices.

A useful indicator for monitoring improvements in implementation of the IPTp policy could be the reduction in missed opportunities for coverage amongst those who could have received it. With the limited impact of demand side factors and the need to focus on the challenges with the primary delivery system, such an indicator may then reflect supply side constraints. However, research needs to be carried out to determine the usefulness of such an indicator and its sensitivity to demand and supply side changes.

In conclusion, this study found low coverage of IPTp in the study area which supported findings that high ANC attendance does not guarantee high IPTp coverage. Demand side factors such as attendance to antenatal care, appropriate timing of attendance, and perceptions about side effects were not significant constraining factors to increased coverage. The scenario is worsened by the reported low experience of directly observed treatment strategy amongst respondents. Further research is required to explore supply side influences to provide better understanding for the low coverage with IPTp.

## Competing interests

The authors declare that they have no competing interests.

## Authors' contributions

CO, KH and OO designed the study. CO and OO were involved in data collection and analysis. CO wrote the initial draft of the manuscript and all the authors participated in its finalization. All authors read and approved the final manuscript.
